# Editorial: Novel immunotherapies to treat gastrointestinal solid tumor cancers

**DOI:** 10.3389/fonc.2022.1043615

**Published:** 2022-10-24

**Authors:** Sripathi M. Sureban, Vita Golubovskaya

**Affiliations:** ^1^ Department of Internal Medicine, The University of Oklahoma Health Sciences, Oklahoma City, OK, United States; ^2^ Promab Biotechnologies, Richmond, CA, United States

**Keywords:** gastrointestinal cancer (GI cancer), immunotherapies and vaccines, clinical trials, PD-1 - PD-L1 axis, solid tumors

Gastrointestinal (GI) solid tumor cancers include cancers of the colorectal, liver, pancreas, gastric, and esophagus. There is an unmet medical need to treat GI cancers as they are the leading cause of cancer-related deaths worldwide (1). Traditional therapies include chemo and radiotherapy, and targeted therapies that do not eliminate GI cancers (2). Novel immunotherapies that target checkpoint inhibitors and reactivation of the host’s anti-tumor immunity to target and treat cancers have been gaining importance (3). Immunotherapies to treat GI cancers are an emerging field of research and are of utmost importance (1, 3). These data taken together provide the strongest rationale to initiate and complete a special issue entitled: Novel Immunotherapies to Treat Gastrointestinal Solid Tumor Cancers

The special issue was focused on identifying novel immunotherapies that are effective against GI cancers. Immunotherapies to treat GI cancers present challenges due to resistance to immune checkpoints and hostile tumor microenvironments. We aimed to provide a spotlight on research involving research-related immunotherapies to treat GI tumors. This special issue includes 12 articles on this topic.

## Original non-clinical research studies

This Research Topic within the Frontiers in Oncology journal has an accumulation of various interesting original research articles. Some of them are so unique that they provide a strong focus on novel and interesting topics.

Wang et al., have focused on a novel approach of conjugating PD-L1 polypeptide (PPA1) with Doxorubicin (DOX) that alleviated resistance to chemotherapy thereby enhancing an anti-colon cancer immune response. This study for the first time provided a dual-functional conjugate of PD-L1 peptide and DOX for cancer cell-targeted drug delivery and inhibition of PD-1 and PD-L1 immune checkpoint and DOX-induced cancer cell death with reduced toxicity. The novelty of this study seems the demonstration of a combined anti-cancer immune response and reduced drug resistance following the treatment with PPA1-DOX.

Wang et al., provided novel evidence that loss of hyaluronan and proteoglycan link protein-1 (HAPLN1), a component of the tumor microenvironment within the extracellular matrix, induces tumorigenesis in colorectal cancers (CRC). Earlier studies have suggested that HAPLN1 plays an important role in cancer tumor stem/progenitor cells under the control of the Wnt signaling pathway (4). In this article, the authors found that expression HAPLN1 was downregulated in CRC tissues when compared with the normal adjacent tissues. HAPLN1 expression was downregulated following TGF-b stimulation and overexpression of HAPLN1 resulted in reduced tumor growth and cancer cell migration *via* inhibition of collagen alpha-1 protein. Their findings suggest that HAPLN1 controls the TGF-β signaling pathway and collagen deposition *via* the TGF-β signaling pathway. This mediates E-adhesion to control tumor growth. The outcome of this study indicates that treatments involving the induction of HAPLN1 levels may represent a novel approach to treating CRC. (Wang et al.) This is study provides novel and compelling evidence of the role of HAPLN1 that is against the current norm. It is unique as the emerging new roles can play a vital role in devising strategies targeting HAPLN1. Also, this study demonstrates that HAPLN1 regulates collagen which is known to regulate immune cell motility (5) providing novel avenues to further investigate the effect of overexpression of HAPLN1 on immune modulation in CRC.

Another important study within this special issue is an efficacy study by Smith et al., where they have demonstrated that the gastrin vaccine treatment alone and with an immune checkpoint antibody inhibits gastric cancer growth and metastases. They have used an immunocompetent syngeneic mouse model of gastric cancer to demonstrate the efficacy of the gastrin vaccine (polyclonal antibody stimulator – PAS) with or without PD-1 antibody. Tumor growth and metastases were significantly inhibited following treatment either with PDS alone or in combination with PD-1 antibody and increased tumor CD8+ T-lymphocytes and decreased immunosuppressive M2-polarized tumor-associated macrophages *via* a gastrin-CCK-BR (cholecystokinin-B receptor) signaling pathway. This compelling study is further validation of the use of the gastrin vaccine as a treatment strategy against gastric cancer.

## Original clinical and retrospect studies

Pan et al., has provided a comprehensive study demonstrating derived neutrophil-lymphocyte ratio (dNLR) with platelet-lymphocyte ratio (PLR) as a potential composite biomarker to identify or correlate the outcomes following the treatment of advanced gastric cancer patient with anti-PD-1 antibodies. Based on the retrospective data from 238 patients with advanced gastric cancer and a significant correlation between the dNLR and PLR, they concluded that their study demonstrates that the combination of dNLR and PLR is a composite biomarker and an independent prognostic factor to evaluate the survival of gastric cancer patients treated with anti-PD-1. They also conclude that the patients with intermediate/poor dNLR/PLR may not benefit from anti-PD-1 treatments. Furthermore, these outcomes need to be further investigated in a larger study. These findings taken together provide novel insights into the predictive efficacy and potentially personalizing immunotherapy for advanced gastric cancer patients. This study provides novel insights into the use of dNLR/PLR as a predictive biomarker to evaluate the efficacy of anti-PD-1 or perhaps other immune therapies.

Cho et al., provided strong clinical evidence of the cost-effectiveness way to treat hepatocellular carcinoma (HCC) with adjuvant immunotherapy using cytokine-induced killer cells.They built a partitioned survival model comparing the expected costs, life-year, and quality of life-year of a hypothetical population of 10,000 patients between cytokine-induced killer (CIK) cell treatments and controls (no adjuvant therapy groups). They concluded that, based on their extensive analysis, HCC patients treated with adjuvant CIK cell was more cost-effective than controls.A further extensive evaluation of other types of immunotherapy needs to be compared to validate this comprehensive study.

In another retrospective study by Sun et al., evidence of the safety and efficacy of treatments with Fruquintinib and PD-1 inhibitors (FP) versus Regorafenib and PD-1 inhibitors (RP) in metastatic colorectal cancer (mCRC). According to their study, the disease control rate and progression-free survival (PFS) were better in the FP group, however, the overall response rate was better in the RP group. Benefits from the FP treatment were observed in patients with no liver metastasis, KRAS wild type, and left colon tumor. Finally, they conclude that their study indicates that better PFS time of patients with mCRC was observed following FP when compared with RP treatments. Though the PFS is merely 2.5 months more than the RP, this study provides insight into the possible treatment strategies that can be considered for patients with no liver metastasis and KRAS wildtype and further validation of this study should be conducted with increasing the number of patients and from various geographical locations.

A meta-analysis by Gu et al., showcased the safety and efficacy of immune checkpoint inhibitors in advanced esophageal squamous cell carcinoma (ESCC). Seven clinical trials with 1733 patients were included in this analysis. The authors conclude that treatment schedules with immune checkpoint inhibitors as second and beyond second-line therapy can improve response and overall survival of esophageal adenocarcinoma patients with locally advanced or metastatic disease. Although, the authors make a summary that not all oncological outcomes for patients can be improved. This correlative study is a pilot study and provides novel insights into the benefits of certain immunotherapies for treating ESCC.

Nie et al. demonstrated the clinical effect of Sintilimab in patients with advanced gastric cancer. The authors concluded that Sintilimab monotherapy or combination therapy had a median PFS of 2.5 months and provides a feasible therapeutic strategy for gastric cancer patients. The study provided the basis for future development therapy of Sintilimab for gastric cancer. This is a good retrospective study that evaluated the clinical efficacy and potential avenue to use Sintilimab in combination with other immunotherapies.

Zhou et al. showed the safety and efficacy of Camrelizumab along with XELOX with bevacizumab or regorafenib in patients with mCRC. This study is based on the study populations with microsatellite-stable mCRC. This is a pilot study and further evaluation with a larger patient population from a diverse geographical location is warranted.

Zhang et al. demonstrated an analysis of the cost-effectiveness of treatments between Cambrelizumab vs placebo added to chemotherapy as primary/first-line therapy in patients with metastatic ESCC. This analysis suggests that the addition of Camrelizumab to chemotherapy is not cost-effective in patients with advanced ESCC in China.

Hong et al. showed data on neoadjuvant immunotherapy combined with chemotherapy followed by surgery versus surgery alone for advanced esophageal squamous carcinoma patients. The authors conclude that this therapy is safe and effective. Further validation of this study is needed with multicentered prospective trials.

## Review article

The review of Huang et al., summarizes data on neoadjuvant therapy for locally advanced esophageal cancers. The authors describe research combining immunotherapy with adjuvant therapy and conclude that for the best synergistic effect future research should be focused on the sequence of immunotherapy and radio(chemo)therapy and biomarkers. This is a very good review article showcasing the development of neoadjuvant therapy for locally advanced esophageal cancers and unsolved clinical problems.

## Conclusions

We tried to provide a comprehensive and diverse type of original research articles in the field of immunotherapies to treat gastrointestinal cancers. This issue provides the current trend of ongoing animal-based research and cost-effective clinical studies and provides novel insights into the potential personalized immunotherapies. The combination of these articles also provides the best strategies for overcoming challenges in treating cold and metastatic GI cancers. Some of the studies also provide novel pathways and future directions in successfully planning to treat GI cancers.

## Author contributions

All authors listed have made a substantial, direct, and intellectual contribution to the work and approved it for publication.

## Conflict of interest

SS has ownership interests in COARE Biotechnology Inc., and is an inventor on multiple patents.

The remaining author declares that the research was conducted in the absence of any commercial or financial relationships that could be construed as a potential conflict of interest.

## Publisher’s note

All claims expressed in this article are solely those of the authors and do not necessarily represent those of their affiliated organizations, or those of the publisher, the editors and the reviewers. Any product that may be evaluated in this article, or claim that may be made by its manufacturer, is not guaranteed or endorsed by the publisher.
